# Endophthalmitis

**DOI:** 10.1111/1469-0691.12118

**Published:** 2013-02-25

**Authors:** M L Durand

**Affiliations:** 1 Infectious Disease Service, Massachusetts Eye and Ear InfirmaryBoston, MA, USA; 2 Department of Medicine, Massachusetts General HospitalBoston, MA, USA; 3 Harvard Medical SchoolBoston, MA, USA

**Keywords:** Bacterial endophthalmitis, *Candida* endophthalmitis, endogenous endophthalmitis, endophthalmitis, fungal endophthalmitis, keratitis-related endophthalmitis, mould endophthalmitis, post-injection endophthalmitis, postoperative endophthalmitis, post-traumatic endophthalmitis

## Abstract

Endophthalmitis means bacterial or fungal infection inside the eye involving the vitreous and/or aqueous humors. Most cases are exogenous and occur after eye surgery, after penetrating ocular trauma, or as an extension of corneal infection. An increasing number of cases are occurring after intravitreal injections of anti-vascular endothelial growth factor (VEGF) medications. Endophthalmitis may also be endogenous, arising from bacteraemic or fungaemic seeding of the eye. The infected eye never serves as a source of bacteraemia or fungaemia, however. The most common pathogens in endophthalmitis vary by category. Coagulase-negative staphylococci are the most common causes of post-cataract endophthalmitis, and these bacteria and viridans streptococci cause most cases of post-intravitreal anti-VEGF injection endophthalmitis, *Bacillus cereus* is a major cause of post-traumatic endophthalmitis, and *Staphylococcus aureus* and streptococci are important causes of endogenous endophthalmitis associated with endocarditis. In Taiwan and other East Asian nations, *Klebsiella pneumoniae* causes most cases of endogenous endophthalmitis, in association with liver abscess. Endogenous fungal endophthalmitis in hospitalized patients is usually caused by *Candida* species, particularly *Candida albicans*. Acute endophthalmitis is a medical emergency. The most important component of treatment is the intravitreal injection of antibiotics, along with vitrectomy in severe cases. Systemic antibiotics should be used in cases of endogenous endophthalmitis and exogenous fungal endophthalmitis, but their role in exogenous bacterial endophthalmitis is uncertain. Repeated intravitreal injections of antibiotics may be necessary if there is no response to the initial therapy. Many eyes that receive prompt and appropriate treatment will recover useful vision.

## Introduction

Endophthalmitis means bacterial or fungal infection inside the eye, involving the vitreous and/or aqueous humors. Most cases of endophthalmitis are exogenous, and organisms are introduced into the eye via trauma, surgery, or an infected cornea. Endogenous endophthalmitis occurs when the eye is seeded via the bloodstream. Patients usually have symptoms from their underlying systemic infection, but sometimes present only with eye symptoms.

Endophthalmitis does not serve as a source of bactaeremia or fungaemia. Infection remains confined to the eye. In panophthalmitis, infection spreads from the globe of the eye to the adjacent soft tissues of the orbit.

Most cases of endophthalmitis present acutely, with hours to a few days of symptoms. These cases are medical emergencies, as delay in treatment may result in permanent vision loss.

## Types of Endophthalmitis

Endophthalmitis may be divided into several categories (Table 1). Regardless of category, treatment requires the prompt intra-ocular injection of antibiotics and, in some cases, a vitrectomy as well.

**TABLE 1:** Types of endophthalmitis, common pathogens, and treatment

Type	Most common pathogens	Intial intravitreal treatment^a^	Vitrectomy necessary^c^	Need to remove artificial intra-ocular lens?	Initial systemic antibiotics^b^
Acute post-cataract	Coagulase-negative staphylococci (70% of cases), other Gram-positive cocci (25%)	Intravitreal vancomycin plus ceftazidime	Yes, if severe infection or fungal aetiology	No, unless fungal aetiology	Value unknown, rarely given
Chronic post-cataract	*Propionibacterium acnes*	Intravitreal vancomycin	Varies	Yes	No
Post-injection	Coagulase-negative staphylococci, viridans streptococci	Intravitreal vancomycin plus ceftazidime	Yes, if severe infection	No	Moxifloxacin or similar?
Bleb-related	Streptococci, *Haemophilus influenzae*	Intravitreal vancomycin plus ceftazidime	Most cases	No	Moxifloxacin or similar?
Post-traumatic	*Bacillus cereus*, coagulase-negative staphylococci (fungi in some cases)	Intravitreal vancomycin plus ceftazidime (plus amphotericin if fungi suspected)	Most cases	Varies (always if fungal)	Intravenous vancomycin plus either ceftazidime or ciprofloxacin
Endogenous bacterial	*Staphylococcus aureus*, streptococci, Gram-negative bacilli (e.g. *Klebsiella*)	Intravitreal vancomycin plus ceftazidime (or amikacin)	Yes, nearly all cases	No	Intravenous antibiotics tailored to systemic infection
*Candida*	*Candida* species	Intravitreal amphotericin (or voriconazole)	Yes, if vitritis	Often	Yes
Mould	*Aspergillus*, *Fusarium*	Intravitreal amphotericin	Yes	Yes	Yes

a Intravitreal antibiotics are given at the end of a vitrectomy case in the operating room, or as an office procedure without a vitrectomy (see text). Whereas initial therapy is empirical, subsequent injections may be tailored to culture results.

b Systemic antibiotics alone are not effective in treating endophthalmitis, except for most cases of *Candida chorioretinitis* without vitritis. They are indicated in endogenous endophthalmitis and fungal endophthalmitis. Whether they are beneficial as adjunctive therapy in exogenous bacterial endophthalmitis is unknown (see text).

c See text for exceptions.

### Acute post-cataract endophthalmitis

Cataract surgery is one of the most common eye operations performed worldwide, and acute post-cataract endophthalmitis complicates this procedure in *c*. 0.1% of cases 1–7. In the USA and Europe, nearly all cases are caused by bacteria, whereas in tropical regions such as India, 10–20% of cases are caused by fungi 8.

Ocular surface bacteria contaminate the aqueous humor in 7–43% of cataract operations, but endophthalmitis is rare 9–11. This may be because the aqueous humor has a rapid turnover time (100 min). The vitreous humor does not regenerate, so it is more susceptible to infection. During cataract surgery, the lens pulp is removed but the posterior lens capsule is left intact. Inadvertent breaks in this capsule increase the endophthalmitis risk 14-fold 6,12. Other endophthalmitis risk factors include clear corneal incision and silicone rather than acrylic intra-ocular lenses (IOLs) 13.

Symptoms occur within 1 week postoperatively in 75% of cases, and include decreased vision (95%), red eye (80%), and eye pain (75%) 14. Patients feel otherwise well and are afebrile. On physical examination, vision is decreased, eyelids are normal to slightly swollen, the conjunctiva is injected, and a hypopyon is present in >80% of cases 15. There are white blood cells in the aqueous humor and vitreous humor, so the view of the retina is hazy. In 80% of patients, the view is so obscured that retinal vessels cannot be seen 16.

#### Diagnosis

Endophthalmitis is a clinical diagnosis, supported by culture of intra-ocular fluids, although a negative culture occurs in 30% of cases. The differential diagnosis is sterile intra-ocular inflammation. This may occur as a reaction to surgery, and is typically greatest on the first postoperative day, whereas endophthalmitis usually occurs on day 2 or later. Toxic anterior segment syndrome, another sterile inflammatory condition, is associated with instrument-cleaning problems or with solutions used during surgery 17,18.

The vitreous humor is sampled through either a vitrectomy or a needle aspirate; the aqueous humor is sampled through a needle aspirate. Needle aspirates can be performed as an office procedure. A vitrectomy is performed in the operating room with a vitrector, which cuts and aspirates the vitreous humor while this is being replaced by saline. The result is *c*. 100 mL of dilute vitreous washings, which may be vacuum-filtered through a 0.45-μm filter; the filter paper can then be cultured. Gram stains are positive in 40–50% of cases. Gram stains may reveal pigment granules from the iris or retinal pigment epithelium, and these may resemble Gram-positive cocci but are more refractile. Vitreous cultures are more likely to be positive after vitrectomy than vitreous aspirate (90% vs. 75%); aqueous cultures are positive in 40% 16. Only the aqueous humor is positive in 4% 19.

Molecular diagnostic techniques have been used to improve the sensitivity of pathogen detection. A multicentre European study of over 16 600 cataract operations evaluated 29 endophthalmitis cases by both culture and PCR, and found that PCR increased the detection of pathogens from 14 to 20 20. In a study from France of 100 post-cataract endophthalmitis cases, the sensitivities of PCR and culture were similar with initial intra-ocular samples, before antibiotics had been injected 21. However, PCR was much more sensitive than culture (70% vs. 9%) in subsequent vitrectomy samples, reflecting either the inhibitory effect of the previously injected antibiotics, or the fact that PCR does not distinguish living from dead organisms. In a study from Brazil of 11 patients with endophthalmitis, cultures were positive in 75% and PCR was positive in 91% 22. This study also tried to evaluate the specificity of PCR; none of the 12 vitreous controls were positive, but two of 50 aqueous samples were. A study from Japan also found improved sensitivity with PCR: of 19 patients with endophthalmitis, 18 were positive by PCR and only ten by culture 23. Control samples were positive by PCR in 6% of 50 patients with uveitis, although in none of 40 patients without intra-ocular inflammation.

#### Microbiology

The major pathogens are coagulase-negative staphylococci (70%), *Staphylococcus aureus* (10%), streptococci (9%), other Gram-positive cocci, including enterococci and mixed bacteria (5%), and Gram-negative bacilli (6%). The fact that Gram-positive bacteria cause >95% of cases reflects the usual pathogenesis, i.e. contamination of the aqueous humor with surface bacteria during surgery. In tropical regions such as India, fungi may cause 10–15% of postoperative endophthalmitis cases.

#### Treatment

The most important component of therapy is the direct injection of antibiotics into the eye. The injection is performed after a sample of vitreous humor is taken for Gram staining and culture. Initially, broad-spectrum antibiotics are used empirically: intravitreal vancomycin 1 mg/0.1 mL normal saline plus either ceftazidime 2.25 mg/0.1 mL or amikacin 0.4 mg/0.1 mL. If there is no improvement in 48 h, a repeat intravitreal injection may be given with either vancomycin or ceftazidime, depending on culture results. Repeated injections of amikacin are avoided, owing to concerns about retinal toxicity.

The second component of treatment is vitrectomy. Vitrectomy surgically debrides the vitreous humor, similarly to draining an abscess, and is the fastest way of clearing infection in eyes with fulminant endophthalmitis. However, vitrectomy is performed in the operating room, whereas a needle aspirate (for culture) is performed in the office: intravitreal antibiotics are given at the end of a vitrectomy case or after vitreous aspirate. A multicentre prospective trial, the Endophthalmitis Vitrectomy Study (EVS), tried to answer the question of whether a vitrectomy was necessary in the treatment of post-cataract endophthalmitis, prior to intravitreal injection of antibiotics 14. The EVS randomized 420 patients into ‘tap’ (needle aspirate or biopsy of vitreous humor) and vitrectomy groups, with both groups receiving intravitreal antibiotics after the procedures 14. Vitrectomy decreased the rate of severe vision loss from 47% (tap group) to 20% (vitrectomy group) in patients who presented with the worst vision (light perception only). No apparent benefit was seen in patients who presented with hand motion or better vision, although the study design may have obscured a possible benefit: two-thirds of patients in the ‘tap’ group actually received a vitreous ‘biopsy’ in the operating room, with a vitrector. We favour vitrectomy for all patients who present with severe vision loss or rapidly worsening vision, or who are likely to have endophthalmitis caused by virulent bacteria such as streptococci.

Systemic antibiotics are not effective in treating endophthalmitis: intravitreal antibiotics must always be given. Whether systemic antibiotics provide any additional benefit to intravitreal antibiotics is unknown. The EVS also tried to answer this question, but the choice of systemic antibiotics used in the study, amikacin plus ceftazidime, was poor 14. These antibiotics have poor activity against staphylococci, the cause of 80% of post-cataract endophthalmitis cases, and amikacin does not cross the blood–eye barrier, and so reaches minimal levels in the vitreous humor.

#### Visual outcome

The visual outcome is highly correlated with the bacteriology. Streptococci of any type produce severe endophthalmitis with a poor chance of visual recovery, whereas coagulase-negative staphylococci cause milder endophthalmitis in general. Overall, 50% of eyes with post-cataract endophthalmitis recover 20/40 vision, and 10% are left with no useful vision (5/200 or less).

### Chronic post-cataract endophthalmitis

Chronic post-cataract endophthalmitis is usually caused by *Propionibacterium acnes*, and presents as a persistent low-grade inflammation in the anterior chamber. Patients present with decreased vision in the affected eye, and half also have eye pain, which is usually mild. On examination, there are white blood cells in the aqueous humor, sometimes a hypopyon, a characteristic white plaque on the posterior lens capsule, and usually inflammation in the anterior vitreous humor.

Intra-ocular cultures may be negative, although culture of the white capsular plaque is often positive. Treatment with a combination of removal or exchange of the IOL, total capsulectomy, vitrectomy and intravitreal antibiotics (e.g. vancomycin) has been the most successful approach. Treatment regimens that leave the original IOL in place have a 40–50% relapse rate.

In rare cases, this entity is caused by fungi, and treatment is different (see below).

### Post-injection endophthalmitis

Intravitreal injections of anti-vascular endothelial growth factor agents (e.g. bevacizumab, ranibizumab, and pegaptanib) are given to treat neovascular macular degeneration. Injections may be repeated monthly for several months, and each injection carries a small risk of causing endophthalmitis. The number of cases of post-injection endophthalmitis is increasing as the use of this treatment increases, and at a tertiary eye centre in Australia there were more cases of post-injection endophthalmitis than of post-cataract endophthalmitis between 2007 and 2010 24. The rate of endophthalmitis per injection is low, with reported rates varying from 0.025% to 0.2% 25–28. A study using a Medicare database of 40 903 injections found an endophthalmitis rate of 0.09% per injection 29.

Symptoms are eye pain and decreased vision, and examination reveals vitreal inflammation. In one large study, all 23 patients with presumed endophthalmitis had pain and vitritis developing 1–6 days after injection (average: 3.4 days), and 78% had hypopyon 28. Cultures were positive in only seven cases. A study in England of 47 post-injection endophthalmitis cases found that patients presented an average of 5 days after injection (range: 1–39 days), and positive cultures occurred in 60% 25.

Gram-positive bacteria cause >95% of culture-positive cases 30. Coagulase-negative staphylococci cause *c*. 60% of cases and viridans streptococci cause 25% 24,30. This is a much higher rate of streptococcal endophthalmitis than in post-cataract cases. Viridans streptococci (*Streptococcus mitis*/*Streptococcus oralis*) also caused an outbreak of 12 cases of post-injection endophthalmitis in Florida, owing to contamination of bevacizumab syringes made up by a single compounding pharmacy 31. Eleven of the 12 patients were left with minimal vision, count fingers, or worse.

Diagnosis and treatment are the same as for post-cataract endophthalmitis (see above). Visual outcomes are poor in cases caused by streptococci.

### Bleb-related endophthalmitis

A filtering bleb is a treatment for glaucoma. It is a surgically created scleral defect, covered only with conjunctiva, that allows excess aqueous humor to be absorbed into the systemic circulation. Because only conjunctiva separates the ocular surface flora from the aqueous humor at the bleb, endophthalmitis may occur at any time. The risk of endophthalmitis is *c*. 1.3% per patient-year 32. One study reported an average onset at 2 years postoperatively (range: 1 month to 8 years) 33. Streptococci, including *Streptococcus pneumoniae*, cause 50% of cases. *Haemophilus influenzae*, *Moraxella catarrhalis*, *S. aureus* and coagulase-negative staphylococci are other pathogens 33.

Vitrectomy and intravitreal antibiotics (e.g. vancomycin plus ceftazidime) are indicated. The value of a systemic agent as adjunctive therapy has not been studied, but it seems reasonable to give an oral quinolone, such as moxifloxacin, that achieves good vitreous levels and treats the major pathogens. The visual outcome varies, depending partly on the pathogen: 40% achieve 20/40 vision or better, whereas 30% lose all vision in the affected eye 34.

### Post-traumatic endophthalmitis

Endophthalmitis occurs in 3–10% of cases after penetrating trauma to the eye, although early surgical repair and prophylactic systemic antibiotics may reduce this incidence to <1% 35. Risk factors for the development of endophthalmitis include metal rather than glass or blunt trauma injuries, retained intra-ocular foreign bodies, disruption of the lens, and delay in primary repair of >24 h.

*Bacillus cereus* is a major pathogen in post-traumatic endophthalmitis, and causes a fulminant infection with very poor visual outcome 36. Onset of symptoms (eye pain, red eye, and decreased vision) usually occurs within 12–24 h of the injury. Findings include marked intra-ocular inflammation and often a ring corneal infiltrate. Other causes of post-traumatic endophthalmitis are coagulase-negative staphylococci, streptococci, Gram-negative bacilli such as *Klebsiella* and *Pseudomonas*, and moulds 37,38.

Treatment should be aggressive, with vitrectomy, intravitreal antibiotics (e.g. vancomycin plus ceftazidime), and systemic therapy.

### Endogenous bacterial endophthalmitis

Endogenous bacterial endophthalmitis arises from bacteraemic seeding of the eye. Endocarditis, usually caused by *S. aureus* or streptococci, accounts for 40% of endogenous endophthalmitis cases in the USA, and other cases are associated with urinary tract infections, indwelling central venous catheters, illicit injection drug use, and procedures such as endoscopy that can cause transient bacteraemia. In Taiwan, Singapore, and other East Asian nations, liver abscess caused by *Klebsiella pneumoniae* is the source of 60% of cases of endogenous endophthalmitis 39.

Many patients present with eye symptoms (pain and blurred vision) rather than symptoms of their underlying infection; this was true of half of the patients in one study 40. Blood cultures are positive in 75% of cases, as are vitreous cultures 40. Common pathogens in western countries include *S. aureus* (25%), streptococci (30–50%, primarily *Streptococcus pneumoniae*, *Streptococcus milleri* group, and group A and B streptococci), and Gram-negative bacilli such as *Escherichia coli* (30%). In Asia, *K. pneumoniae* causes the majority of cases.

Treatment of the underlying source of bacteraemia with systemic antibiotics is necessary, but this will not effectively treat the endophthalmitis: intravitreal antibiotics and, usually, a vitrectomy are necessary.

### *Candida* endophthalmitis

#### Endogenous *Candida* endophthalmitis

Most cases of *Candida* endophthalmitis are endogenous, occurring as a complication of candidaemia. The candidaemia usually occurs in hospitalized patients, often as a complication of an indwelling central venous catheter. Other risk factors include total parenteral nutrition, broad-spectrum antibiotics, neutropenia, recent abdominal surgery, and glucocorticoid therapy.

Candidaemia seeds the highly vascular choroid first, so the initial manifestation is usually chorioretinitis or choroiditis. There may be minimal or no vitreous inflammation at first. Infection in the choroid and retina is often painless, so, unless the lesions are near the macula, patients with early *Candida* chorioretinitis may have no symptoms. As the infection worsens, vitritis develops, and patients develop decreased vision. With inflammation spreading to the aqueous humor, eye pain may also be a presenting symptom.

Often, the literature distinguishes *Candida* chorioretinitis from *Candida* endophthalmitis, the latter term being reserved for those cases with significant vitritis. A term that includes both *Candida* chorioretinitis and endophthalmitis (i.e. vitritis) is ocular candidiasis.

The prevalence of ocular candidiasis in candidaemia must be evaluated by prospective studies. In such studies, the incidence varies from 2% to 26%, with most cases being attributable to chorioretinitis and only 0–6% having significant vitritis (i.e. ‘endophthalmitis’) 41–44. In a multicentre trial involving 370 patients with candidaemia, possible or probable ocular involvement occurred in 16%, with most having chorioretinitis as the manifestation and only 1.6% being classified as having endophthalmitis (i.e. with significant vitritis) 41.

Outpatients may also present with endogenous *Candida* endophthalmitis, and some of these patients have a recent history of hospitalization and a recent indwelling central venous catheter. However, most outpatients who present with *Candida* endophthalmitis have a history of illicit intravenous drug use. In a study from Australia, 70% of 27 patients presenting with fungal endophthalmitis between 2001 and 2007 were injection drug users 45. Candidaemia is often transient in injection drug users and in patients with recent indwelling central venous catheters, so patients may present with decreased vision as their only symptom. They may be misdiagnosed initially as having a sterile uveitis, unless a history of intravenous drug use or recent hospitalization is obtained.

The diagnosis of ocular candidiasis is made on the basis of eye examination and confirmed with vitreous culture. Vitreous culture is usually not necessary in patients who have known candidaemia and eye findings compatible with chorioretinitis. Funduscopic examination shows white fluffy lesions in the choroid or retina, sometimes with vitritis (endophthalmitis). There may be so much vitritis that the retina may be obscured. A ‘string of pearls’ or ‘snowballs’ may be seen in the vitreous humor, representing clumps of infection within the inflamed vitreous humor. In outpatients who present with eye findings but do not have a history of known candidaemia, diagnosis must be suspected by clinical appearance (fluffy white infiltrates in the retina or vitreous humor, often with marked vitritis), and confirmed by vitreous culture.

*Candida albicans* is the most common cause of ocular candidiasis, causing 92% of cases in one review; *Candida tropicalis* was the second most common cause 46.

All patients with ocular candidiasis must be followed closely by an ophthalmologist, as some patients worsen despite therapy. In a multicentre study of 370 patients with candidaemia who received either voriconazole or amphotericin followed by fluconazole, 18% of the 60 ocular candidiasis cases developed while antifungal therapy was being given 41. For most patients with chorioretinitis alone, the lesions will respond to the systemic antifungal therapy. Patients who have significant vitritis (endophthalmitis) require a vitrectomy and intravitreal amphotericin (or voriconazole), in addition to systemic antifungal therapy with an agent that crosses the blood–eye barrier, such as fluconazole (if susceptible), voriconazole, or amphotericin.

#### Exogenous *Candida* endophthalmitis

Exogenous cases are rare, and most cases occur following surgery. Inflammation may initially be greatest in the anterior chamber, before involving the vitreous humor.

*Candida parapsilosis* is the most common species, especially in postsurgical outbreaks. The reason is most likely that this species appears to survive well in irrigation fluids and on prosthetic materials 47. An outbreak in the USA in 1983 affecting 13 patients in different states was traced to a balanced salt solution used during ophthalmic surgery that was contaminated during manufacture 48.

Treatment includes intra-ocular injection of either amphotericin or voriconazole, vitrectomy, and systemic voriconazole or fluconazole (if the organism is susceptible). The dose of intravitreal amphotericin is 5–10 μg in 0.1 mL of sterile water, and the dose of intravitreal voriconazole is usually 100 μg in 0.1 mL of sterile water. If infection follows cataract surgery, it is often necessary to remove the IOL as well. Systemic therapy with high-dose fluconazole (400–800 mg daily, assuming normal kidney function) is also indicated for susceptible strains, or voriconazole for fluconazole-resistant but voriconazole-susceptible strains.

### Mould endophthalmitis

Two-thirds of patients with mould endophthalmitis lose useful vision, but the prognosis may be improving with the use of newer antifungal agents.

#### Exogenous mould endophthalmitis

Mould endophthalmitis is usually exogenous, occurring after eye surgery, after eye trauma, or as a result of keratomycosis (fungal corneal infection). *Aspergillus* and *Fusarium* are the most common aetiological agents.

Postoperative mould endophthalmitis is rare in western countries. When it does occur, it often presents subacutely, and diagnosis may be delayed. In one review that included ten cases of postoperative mould endophthalmitis, 60% presented ≥2 months after surgery 49. In tropical regions such as India, moulds have caused >20% of postoperative endophthalmitis cases in some series 8,50, and 80% of cases present within 4 weeks of surgery. Outbreaks of postoperative mould endophthalmitis have also occurred.

Post-traumatic mould endophthalmitis is also more common in India than in the USA or Europe. Moulds caused 14% of 113 culture-positive post-traumatic endophthalmitis cases in a series from India 51. In another series from India, the average time to presentation after eye trauma was 7 days 52. *Aspergillus* was the most common mould isolated in both of these series.

Mould infection of the cornea (keratomycosis) may lead to endophthalmitis as the mould grows through the cornea and into the aqueous humor. Keratomycosis was the aetiology for half of all exogenous mould cases in a series from Florida, and eye surgery and trauma each accounted for 25% of cases 49. Many cases of keratomycosis are associated with contact lens wear. *Fusarium* is the most common cause of endophthalmitis resulting from keratomycosis in many series. Some of these cases reflect the international outbreak of *Fusarium* keratitis that occurred in 2004–2006, related to one brand of contact lens cleaning solution. In this outbreak, 6% of keratitis cases developed endophthalmitis 53,54.

The clinical presentation of exogenous mould endophthalmitis may be subacute, as noted above. Slit lamp examination of the eye often shows hypopyon and clumps of thick white material in the anterior chamber, particularly after trauma or surgery. There may be vitritis. When endophthalmitis complicates keratitis, slit lamp examination will show the corneal infection, and often shows filaments extending from the back of the cornea into the aqueous humor.

Diagnosis is made by culture of the aqueous humor as well as the vitreous humor. Treatment requires surgery to clean out thick material, if present, in the aqueous humor, vitrectomy for significant vitritis, intracameral and intravitreal injections of antifungal agents (amphotericin or voriconazole), and systemic voriconazole therapy. Systemic voriconazole achieves very good intra-ocular levels, of *c*. 40% of serum levels. Unless the fungus is known, the initial intra-ocular injection should be amphotericin; subsequent injections may be voriconazole for sensitive fungi. Repeated intra-ocular injections of voriconazole (if the organism is susceptible) or amphotericin can be given, at least 48 h apart. Voriconazole is preferred for susceptible strains, as it appears to be less irritating than amphotericin. If there is an IOL, this must be surgically removed. In cases related to keratomycosis, a corneal transplant is almost always necessary.

Several case reports have described salvage with posaconazole for cases of *Fusarium* keratomycosis-related endophthalmitis in which other therapies have failed 55,56. Itraconazole is not recommended, as it does not achieve good levels in the vitreous humor; and fluconazole should not be given, as it has poor activity against moulds. Systemic amphotericin has significant toxicity, and is rarely used to treat exogenous fungal endophthalmitis, but may be considered as adjunctive therapy in those rare cases of azole-resistant fungal endophthalmitis in which other therapies have failed.

#### Endogenous mould endophthalmitis

Endogenous mould endophthalmitis is rare. It usually occurs in severely immunocompromised patients with fungaemia, or in injection drug users. *Aspergillus* and *Fusarium* are common aetiological agents. Hospitalized patients with mould fungaemia are usually very ill, and may not be able to complain of eye symptoms. In a retrospective study at a Texas cancer centre, only 27% of the 15 patients with mould endophthalmitis survived for >4 weeks 57. Often, the diagnosis of mould endophthalmitis is missed during life. In an autopsy study of 85 orthotopic liver transplant recipients, *Aspergillus* endophthalmitis was noted in 7%, but only one of six patients was diagnosed ante-mortem 58. In contrast, injection drug users who have endogenous mould endophthalmitis are usually well except for their eye, with no evidence of systemic infection. In these patients, fungaemia is usually transient, occurring only at the time of intravenous drug injection.

In immunocompromised patients, treatment must include systemic antifungal therapy, with the choice (e.g. amphotericin or voriconazole) tailored to optimize treatment for the fungaemia. Vitrectomy and removal of any IOL, followed by intravitreal amphotericin or voriconazole (for susceptible strains), should be performed if the patient is able to tolerate surgery. If too ill for surgery, the patient should have intravitreal injection of amphotericin or voriconazole, with repeated injections as needed. In injection drug users with no evidence of ongoing fungaemia, vitrectomy, intravitreal antifungal injection and systemic therapy should be given.

## Conclusion

Endophthalmitis is a medical emergency. The most important component of therapy is intravitreal injection of antibiotics. Vitrectomy is also important in many cases, and leads to improved visual outcomes in patients who present with significant visual loss. Vitrectomy should also be performed in cases that have failed to respond to initial intravitreal injection. Systemic antibiotics are necessary to treat the underlying infection in endogenous endophthalmitis, and as adjunctive therapy in exogenous fungal endophthalmitis. Visual prognosis is partly related to the virulence of the pathogen. The best outcomes usually occur in cases that are either culture-negative or are caused by coagulase-negative staphylococci, and the worst outcomes typically occur in endophthalmitis caused by streptococci, *Bacillus* species, and moulds. Even in cases of severe endophthalmitis caused by virulent pathogens, however, prompt therapy may save useful vision. Even light perception vision is considered to be useful to a blind patient.

## Transparency Declaration

This work was undertaken without funding support or study sponsors. The author received no compensation for the work, and has no relevant conflicts of interest to declare.
